# Spontaneous thought characteristics are differentially related to heightened negative affect versus blunted positive affect in adolescents: An experience sampling study

**DOI:** 10.1002/jcv2.12110

**Published:** 2022-11-22

**Authors:** Christian A. Webb, Anna O. Tierney, Hallie A. Brown, Erika E. Forbes, Diego A. Pizzagalli, Boyu Ren

**Affiliations:** ^1^ Harvard Medical School Boston Massachusetts USA; ^2^ McLean Hospital Belmont Massachusetts USA; ^3^ Department of Psychiatry University of Pittsburgh Pittsburgh Pennsylvania USA

**Keywords:** adolescents, affect, ecological momentary assessment, mind‐wandering, spontaneous thought

## Abstract

**Background:**

Mind‐wandering has been linked to negative affect (NA) and depressive symptoms in adolescents. However, mind‐wandering is an extremely broad and heterogeneous cognitive construct. Some features of spontaneous thought may be related to increased NA, whereas others may improve affect, or have no emotional influence. We used ecological momentary assessment (EMA) to investigate the characteristics of spontaneous thoughts in adolescents and their differential relations with moment‐to‐moment affect.

**Method:**

One‐hundred and sixteen adolescents (ages 13–18; Typical Mood [TM] = 58; Low Mood [LM] = 58) completed 5 days (2–3 times/day) of EMA (total 1037 surveys) assessing current positive affect (PA) and NA and dimensions of spontaneous thought. Multilevel models tested the relation between thought characteristics and affect.

**Results:**

Relative to the TM group, LM adolescents had a higher frequency of mind‐wandering (38% vs. 56%) and negatively‐valanced thoughts during episodes of mind‐wandering (21% vs. 37%). Negatively‐valenced, self‐referential and past‐oriented thoughts were each associated with higher NA, even when controlling for plausible confounds (e.g., engagement in an unpleasant activity or social interaction, depressive symptom severity). In contrast, task‐focused and positively‐valenced thoughts were uniquely linked to higher PA.

**Conclusion:**

Characteristics of spontaneous thought—including temporal orientation, self‐referential quality, and task‐relatedness—were differentially related to NA versus PA in adolescents. If replicated, these findings could inform more nuanced assessments of and targeted interventions for specific dimensions of mind‐wandering contributing to high NA versus blunted PA in teens.

## Graphical abstract

In this study, one–hundred and sixteen adolescents completed ecological momentary assessment (EMA) surveys assessing current positive and negative affect (PA and NA) and dimensions of spontaneous thought. Characteristics of spontaneous thought—including temporal orientation, self–referential quality, and task–relatedness—were differentially related to NA versus PA in adolescents. If replicated, these findings could inform more nuanced assessments of and targeted interventions for specific dimensions of mind–wandering contributing to high NA versus blunted PA in teens.

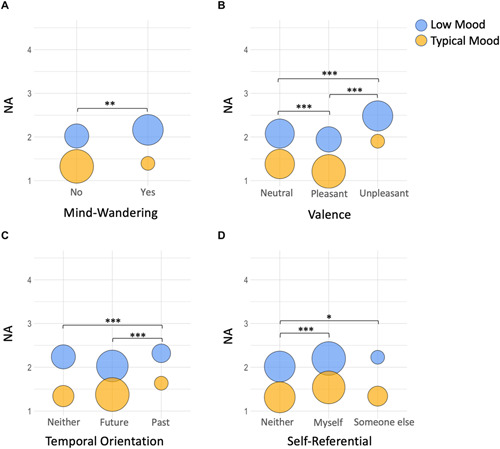

**Key Points**

- Smartphone‐delivered ecological momentary assessment (EMA) provides a useful tool to sample in‐the‐moment thoughts and their relationship to affect in the daily lives of adolescents.
- In this study, one‐hundred and sixteen adolescents completed 5 days of EMA assessing current positive and negative affect (PA and NA) and dimensions of spontaneous thought.
- Characteristics of spontaneous thought—including temporal orientation, self‐referential quality, and task‐relatedness—were differentially related to NA versus PA in adolescents.
- If replicated, these findings could inform more nuanced assessments of and targeted interventions for specific dimensions of mind‐wandering contributing to high NA versus blunted PA in teens.

## INTRODUCTION

We spend a substantial proportion (approximately 30%–60%) of our waking hours mind wandering, or thinking about something other than what we are currently doing (Kane et al., 2007, 2017; Killingsworth & Gilbert, 2010; Webb, Israel et al., 2021; see Seli et al., 2018 for the influence of response options on estimates of mind‐wandering). Although the propensity to mind‐wander may be more common among adolescents relative to children or adults (Carriere et al., 2010; Giambra, 2000; Stawarczyk, 2018; but see Stawarczyk et al., 2014), studies have only recently investigated the content, correlates and consequences of mind‐wandering in teens (e.g., Figueiredo et al., 2020; Fredrick & Becker, 2021; Mrazek et al., 2013; Vannucci et al., 2020; Webb, Israel et al., 2021).

Adolescence represents a period of dramatic physical, social, emotional, and cognitive development (Lam et al., 2014; Mills et al., 2014; Nolen‐Hoeksema & Hilt, 2008; Sawyer et al., 2012, 2018), which contributes to an increasingly rich and complex internal mental world, including frequent mind‐wandering toward developmentally salient content (e.g., reflecting on recent conversations or interpersonal events, mentally simulating and rehearsing upcoming social interactions with peers, social comparisons, and other self‐referential thoughts). The brain networks subserving mind‐wandering and self‐referential thought, in particular the Default Mode Network, undergo significant maturation during the transition from childhood to adolescence (Fair et al., 2008; Sherman et al., 2014). At the same time, the prefrontal circuitry underlying the higher‐order cognitive abilities (e.g., meta‐cognition, attentional control) required to exert control over mind‐wandering does not reach full maturity until well into the twenties (Giedd, 2004). In addition, adolescence is a critical period of self‐concept formation, heightened sensitivity to social context (in particular with regards to peers), and increased self‐referential thinking relative to children or adults (Moses‐Payne et al., 2022; Sebastian et al., 2008). There is also evidence that adolescents, relative to children or adults, have heightened *negative* self‐referential thoughts (Moses‐Payne et al., 2022), which may mean that thoughts about the self may be more likely to elicit NA during this developmental period. Moreover, studies indicate that the ability to reflect on the perspective of others increases during adolescence, including to inform one's own self‐concept (Dumontheil et al., 2010; Moses‐Payne et al., 2021; Pfeifer et al., 2009; Symeonidou et al., 2016). In summary, adolescents may occupy a unique developmental period characterized by an increased propensity to mind‐wander—including towards affectively‐salient thought content—but are still developing their abilities to regulate these mental states.

Adolescence is also the developmental period during which negative affective states increase in frequency (Bailen et al., 2019; Frost et al., 2015; Griffith et al., 2021; Larson et al., 2002) and, relatedly, rates of depression surge (Avenevoli et al., 2015). The prevalence of interpersonal and other (e.g., academic) stressors during the adolescent years (Hammen, 2009; Nolen‐Hoeksema & Hilt, 2008; Rudolph, 2017), coupled with heightened social concerns and a growing capacity for abstract, self‐evaluative thought (Aldao et al., 2010; Blakemore & Mills, 2014; Gaté et al., 2013; Piaget, 1972), may collectively contribute to an increased propensity to mind‐wander towards cognitive content that triggers NA. Given that adolescents spend a substantial proportion of their waking hours mind‐wandering (e.g., approximately 60% according to a recent ecological momentary assessment [EMA] study; Webb, Swords et al., 2021), research is needed to understand the extent to which these states may contribute to increases in NA. Initial research has been mixed, with some studies indicating that mind‐wandering is linked to worse affect in adolescents (Mrazek et al., 2013; Webb, Israel et al., 2021), and others failing to support this relationship (Fredrick & Becker, 2021). These inconsistencies may be due to prior research not distinguishing between different relevant characteristics of spontaneous thought. Mind‐wandering is an extremely broad and heterogeneous cognitive construct (Smallwood & Schooler, 2015; Wang et al., 2018; Welz et al., 2018). Some forms of spontaneous thought may contribute to increased NA (e.g., negatively‐valenced, self‐referential thoughts), whereas others (e.g., daydreaming, future‐oriented anticipatory pleasure) may improve affect or have no affective consequences. Relevant dimensions of thought that may influence affect include the valence of cognitive content (i.e., unpleasant, pleasant, or neutral thoughts), the temporal orientation (i.e., thoughts focused on the past, present or future) (e.g., Ruby et al., 2013; Smallwood & O'Connor, 2011), and the self‐referential quality of thoughts (i.e., focused on the self, others or neither) (Goldstein, 2016; Hadash et al., 2016). For example, rumination can be considered a subset of mind‐wandering that is characterized by repetitive, negatively‐valenced, past‐focused and self‐referential thoughts (for a distinction between rumination and mind‐wandering, see Christoff et al., 2016). Rumination prospectively predicts negative emotional states and depression onset (for reviews see Aldao et al., 2010; Nolen‐Hoeksema et al., 2008; Nolen‐Hoeksema & Watkins, 2011; Watkins & Roberts, 2020), and increases during adolescence (Buerke et al., 2021; Hampel & Petermann, 2005; Sütterlin et al., 2012; Thompson et al., 2010). Relatedly, in a sample of adults, Ruby et al. (2013) reported that past‐oriented thoughts were associated with subsequent low mood, whereas future‐related thoughts were associated with improvement in mood.

In the present study, we used smartphone‐delivered EMA to investigate several relevant characteristics of spontaneous thoughts in adolescents and their differential relations with moment‐to‐moment affect. Relative to conventional retrospective self‐report questionnaires, EMA allows for a more fine‐grained and ecologically valid assessment of fluctuating cognitions and affect in the daily lives of adolescents, while minimizing recall biases by asking youth to report on current thoughts and emotions (Shiffman et al., 2008). In addition, we tested whether dimensions of spontaneous thought may be differentially related to negative affect (NA) versus positive affect (PA), which are only modestly inversely correlated in adolescents (approximate within‐person *r* = −0.30; Herres et al., 2018; Schmidt et al., 2019). Finally, we controlled for several plausible confounds of the relation between mind‐wandering and affect, including current activity, social context, and depressive symptom severity. These variables may serve as third variable confounds in so far as they predict *both* worse affect and an increased likelihood to mind‐wander. In such cases, mind‐wandering may be significantly correlated with worse affect, even if mind‐wandering is not in fact causally related to affect. For example, to the extent that engagement in a relatively unpleasant activity predicts both worse affect and an increased likelihood to mind‐wander, failure to control for such activities would yield a spurious association between mind‐wandering and worse affect (assuming they are in fact not causally related to each other). Similarly, being alone may contribute to relatively poorer affect and promote greater mind‐wandering. Finally, individuals with elevated depressive symptoms may have both worse affect and be more prone to mind‐wandering (e.g., due to ruminative and other task‐unrelated depressogenic thoughts), even if mind‐wandering and worse affect are not causally related.

Informed by prior research, we hypothesized that mind‐wandering would be associated with higher NA and lower PA in adolescents. However, we expected these associations to be no longer significant after controlling for three relevant dimensions of thought (i.e., valence, temporal orientation, and self‐referential quality). We predicted that negatively‐valenced, past‐oriented and self‐referential thoughts would be associated with higher NA, whereas positively‐valenced, future‐oriented, and socially focused cognitions would predict higher PA, even while adjusting for plausible third variable confounds. To increase variability in affect, and given recent evidence of significant differences in the frequency and content of mind‐wandering among adolescents with typical mood (TM) versus low/depressed mood (LM) (Webb, Israel et al., 2021), we recruited a sample of LM adolescents in addition to a TM group. The latter study revealed that LM adolescents had lower PA and both higher NA and rates of mind‐wandering than TM adolescents. The inclusion of both groups increases variance in our predictor and outcome variables, which is necessary for the statistical modeling of their relationships. In addition, it allows for a comparison of the daily (assessed via momentary EMA) affective and cognitive characteristics of a TM group with a sample of teens experiencing depressed affect, which increases in prevalence during the adolescent years.

## METHOD

### Participants

Participants were 116 (TM = 58; LM = 58) 12–18‐year olds with English fluency recruited from the greater Boston area. The following exclusion criteria applied to both groups: history of head trauma with loss of consciousness for 2 min or more; history of seizure disorder; serious or unstable medical illness; current use of dopaminergic drugs, cocaine or stimulant (prescribed or illicit); or clinical or laboratory evidence of hypothyroidism. Given that this study was derived from a larger project which included functional magnetic resonance imaging (fMRI), other exclusion criteria included systemic medical or neurological illness that could impact fMRI measures of cerebral blood flow and other standard exclusion criteria for fMRI scanning (e.g., cardiac pacemaker). Given that data for this study were derived from an ongoing clinical trial for adolescents with elevated anhedonia, participants in the LM group received a score of 3 or higher on the Snaith‐Hamilton Pleasure Scale (SHAPS; original scoring guidelines) (Snaith et al., 1995), and indicated elevated anhedonia (item score >1) on the Schedule for Affective Disorders and Schizophrenia for School‐Age Children Present and Lifetime Version (K‐SADS‐PL) (Kaufman et al., 1997). TM participants had a SHAPS score of 0. The following additional exclusion criteria were applied to participants in the TM group: history of any Diagnostic and Statistical Manual of Mental Disorders, Fifth Edition (DSM‐5) psychiatric or substance‐related disorder, first degree relative diagnosed with Major Depressive Disorder (MDD), bipolar disorder or a psychotic disorder, or current use of any psychiatric medications. For the LM group, history, or current diagnosis of any of the following DSM‐5 psychiatric illnesses were exclusionary: schizophrenia spectrum or other psychotic disorder, bipolar disorder, anorexia nervosa or bulimia nervosa, substance (including alcohol) use disorder within the past 12 months or lifetime severe substance use disorder or chronic depression (current episode ≥2 years). With the exception of obsessive compulsive disorder, all anxiety disorders were permissible.

Demographic and clinical characteristics (including anhedonia and overall depression severity) are presented in Table 1. Note that depression (Center for Epidemiological Studies Depression Scale [CES‐D]) scores ranged from none (0) to severe (51), and 50% (58/116: 55 from the LM group and three from the TM group) of the sample had clinically significant levels of depressive symptoms (CES‐D ≥ 16) (Chwastiak et al., 2002).

**TABLE 1:** Clinical and demographic characteristics of the sample

Sample characteristics					
Low mood	*N*	%	Typical mood	*N*	%
Biological sex			Biological sex		
Female	39	67.2	Female	41	70.7
Male	19	32.8	Male	17	29.3
Race			Race		
American Indian or Alaska Native	0	0.0	American Indian or Alaska Native	0	0.0
Asian	4	6.9	Asian	4	6.9
Black or African American	6	10.3	Black or African American	7	12.1
Native Hawaiian or other Pacific Islander	0	0.0	Native Hawaiian or other Pacific Islander	1	1.7
White	41	70.7	White	41	70.7
Other	1	1.7	Other	0	0.0
More than one race	6	10.3	More than one race	5	8.6
Ethnicity			Ethnicity		
Hispanic or Latino	5	8.6	Hispanic or Latino	4	6.9
Not Hispanic or Latino	53	91.4	Not Hispanic or Latino	54	93.1
Current diagnoses (DSM‐5)			Current diagnoses (DSM‐5)		
Major depressive episode	25	43.1	Major depressive episode	0	0.0
Generalized anxiety disorder	10	17.2	Generalized anxiety disorder	0	0.0
Social anxiety disorder	7	12.1	Social anxiety disorder	0	0.0
Panic disorder	2	3.5	Panic disorder	0	0.0
Specific phobia	1	1.7	Specific phobia	0	0.0
Attention‐deficit/			Attention‐deficit/		
Hyperactivity disorder	4	6.9	Hyperactivity disorder	0	0.0
Oppositional defiant disorder	2	3.5	Oppositional defiant disorder	0	0.0
Medication			Medication		
SSRI	9	15.5	SSRI	0	0.0
	***M***	***SD***		***M***	***SD***
Age (in years)	15.9	1.8	Age (in years)	16.1	1.9
Family income (dollars)	126,000	68,000	Family income (dollars)	179,000	147,000
SHAPS score	34.4	5.8	SHAPS score	18.5	4.5
CES‐D score	33.4	9.9	CES‐D score	5.8	5.0

*Note*: There were no group differences in sex, race, ethnicity, or age (*p*s > .65). There were group differences in SHAPS (*p* < .001) and CESD severity (*p* < .001), as well as income (*p* = .025; 16 subjects did not report their income).

Abbreviations: DSM‐5, Diagnostic and Statistical Manual of Mental Disorders, Fifth Edition; SHAPS, Snaith‐Hamilton Pleasure Scale; SSRI, selective serotonin reuptake inhibitor.

This study is a follow‐up to a prior investigation in a smaller sample which focused on the variable of mind‐wandering and its relation to resting state brain functional connectivity and affect in adolescents with typical mood versus low mood (Webb, Swords et al., 2021). The present study investigates a much broader set of thought characteristics, including mind‐wandering but also the role of thought valence, temporal orientation, and self‐referential quality in relation to affect. Forty‐nine percent (57/116) of the participants in this study were included in the prior study.

### Procedure

The study was approved by the Mass General Brigham IRB. Written informed consent was required from participating parents and children provided assent (18 year olds provided consent). Following an initial screening session, involving self‐report questionnaires (including the SHAPS) and a clinical diagnostic (K‐SADS‐PL) interview, participants completed an MRI session (see Supporting Information S1 for additional details on measures). Participants completed 5 days of EMA smartphone surveys following their MRI scan (prior to the initiation of therapy in the abovementioned clinical trial from which these data were derived), with surveys triggered 2–3 times per day via popup notifications on teen's smartphones using the Metricwire app. These EMA surveys were delivered Thursday through Monday, two to three times per day, in order to sample current affect and cognitions on both weekdays (i.e., school days) and the weekend (For similar EMA designs in adolescents, see Alarcón et al., 2020; Carper et al., 2022; Forbes et al., 2009, Murray et al., 2022; Webb, Swords et al., 2021). EMA surveys were triggered using a time‐stratified random sampling strategy such that adolescents were signaled once at a random time during two timeslots (4 p.m. to 6:30 p.m. and 6:30 p.m. to 9:00 p.m.), with surveys separated by at least 1 h. To not disturb students during school, surveys were outside of school hours on weekdays. An additional survey was sent on weekends between 11 a.m. and 4 p.m. Participants were incentivized to complete a higher proportion of EMA surveys, earning $15 and $22.50 if they completed at least 70% or 80% of surveys, respectively. The mean length of time between EMA surveys within a given day was approximately 3 h (mean 184.7 min; SD = 152.2 min). There were 1037 EMA surveys completed in total. Mean EMA compliance was 76.6% (SD = 21.8%) and 72.4% (SD = 22.8%) for the TM and LM groups, respectively (*t*(114) = 1.01, *p* = .317). There were no significant associations between baseline demographic (age and gender) or clinical (SHAPS and CESD) variables and EMA compliance (% of surveys complete), nor between EMA factors (mean PA, NA and mind wandering %) and EMA compliance (*p*s > .19). In addition, there was no association between how long it took participants to respond to a survey prompt and mind‐wandering (*p* = .45).

### Ecological momentary assessment (EMA) measures

#### Positive and negative affect

At each EMA survey timepoint, participants were asked to rate the extent to which they felt “happy,” “interested,” and “excited,” (PA items) and “sad,” “nervous,” and “angry” (NA items) “right before you started this survey.” Participants gave their responses using a 5‐point Likert scale, with 1 corresponding to “very slightly or not at all,” and 5 corresponding to “extremely.” Their ratings for these two groups of emotions were averaged to obtain their mean PA and NA scores for each timepoint. Split‐half reliabilities for PA (*r* = 0.86) and NA (*r* = 0.88) were high (see Supporting Information S1).

#### Mind‐wandering and related thought characteristics

Participants were asked what they were thinking about “right before you started the survey” and if they were thinking about something other than what they were doing, with an answer of “Yes” being coded as an instance of mind‐wandering. They were then asked: (1) if they were thinking about something that had happened in the past, something in the future, or neither (i.e., *temporal orientation*); (2) if they were thinking about something pleasant, unpleasant, or neutral (i.e., *thought valence*) and (3) if they were thinking about themselves, someone else, or neither/something else (i.e., *self‐referential quality*).

#### Current activity

Participants were asked to write down what they were doing when they received the survey and rate how much they enjoyed that activity using a 5‐point Likert scale ranging from “very slightly or not at all” to “extremely.” Participants also indicated whether they were with someone or alone at the time that they received the survey.

### Data analytic plan

To test group differences in categorical outcomes (i.e., mind‐wandering [yes vs. no], valence [pleasant, unpleasant, vs. neutral thoughts], temporal orientation [thoughts about the past, future vs. present/neither], and self‐referential quality [thoughts about the self, others, vs. neither]) we used a Bayesian hierarchical multinomial logistic model implemented in the R (Vers. 4.1.0) package *brms* (Bürkner, 2017). Each outcome was analyzed separately, and group label (LM vs. TM) was the only predictor in the model (see Supporting Information S1 for additional details).

Next, given the multilevel data structure (i.e., repeated EMA assessments nested within individuals) a multilevel modeling approach was used to test the relation between predictor variables (i.e., thought characteristics) and affect over time. A vector of PA or NA scores for each adolescent participant served as the dependent variable (Time T), with scores on the predictor variable (also at Time T) serving as the independent variable. Separate models were run for PA and NA. We first tested whether mind‐wandering was associated with higher NA and/or lower PA. Next, we tested multivariable models which simultaneously included each of the abovementioned thought characteristics (mind‐wandering, valence, self‐referential quality, and temporal orientation) as predictors. For those variables with more than two possible levels, dummy variables were used to code them. We also included several plausible confounds of the relation between thought characteristics and affect, including depressive symptom severity, current activity pleasure rating and social context (alone or with someone). Finally, lagged analyses tested whether an independent variable predicted PA or NA at the next timepoint. Given the extended length of time between the last EMA survey of day and the first survey the following day, spaces were added in between days so that lagged analyses only considered associations *within* days. Analyses with continuous affect (PA and NA) outcomes were conducted with the *lme4* (Bates et al., 2014) and *lmerTest* (Kuznetsova et al., 2017) packages in R, specifying a random intercept and using an intent‐to‐treat approach (i.e., all subjects included). Time was included as a covariate in all analyses.

To estimate the proportion of variance in PA and NA scores accounted for by our predictors we report the marginal and conditional coefficient of determination (*R* ^2^) for generalized mixed‐effect models. As another approach to interpreting the magnitude of predictor effects, we divided parameter estimates by the mean within‐person SD of NA (0.45) and PA (0.63). For example, assuming analyses reveal a parameter estimate of 0.23 for the association between mind‐wandering and NA (i.e., NA scores are 0.23 higher, on average, when mind‐wandering relative to when one is not mind‐wandering) then the effect size (ES) estimate would be 0.51 (0.23/0.45), meaning that the mind‐wandering—NA relationship is, on average, approximately half the magnitude of the average within‐person SD of NA. The latter ES would be considered a medium effect using guidelines for small (<0.2), medium (0.5), and large (>0.8) effects (Cohen, 1988). Finally, using Monte Carlo simulations (1000 repetitions; alpha = 0.05; SIMR package in R) (see Green & MacLeod, 2016), a power analysis was conducted informed by effect sizes from Webb, Swords et al. (2021), which focused on the relationship between mind‐wandering and affect in adolescents. The current study with a sample of 116 would have a 100% power to detect an effect the same magnitude as that reported for the mind‐wandering‐NA relationship in the latter study (96.8% power to detect an effect three‐quarters that magnitude and 70.2% power to detect an effect half that magnitude).

## RESULTS

**FIGURE 1:**
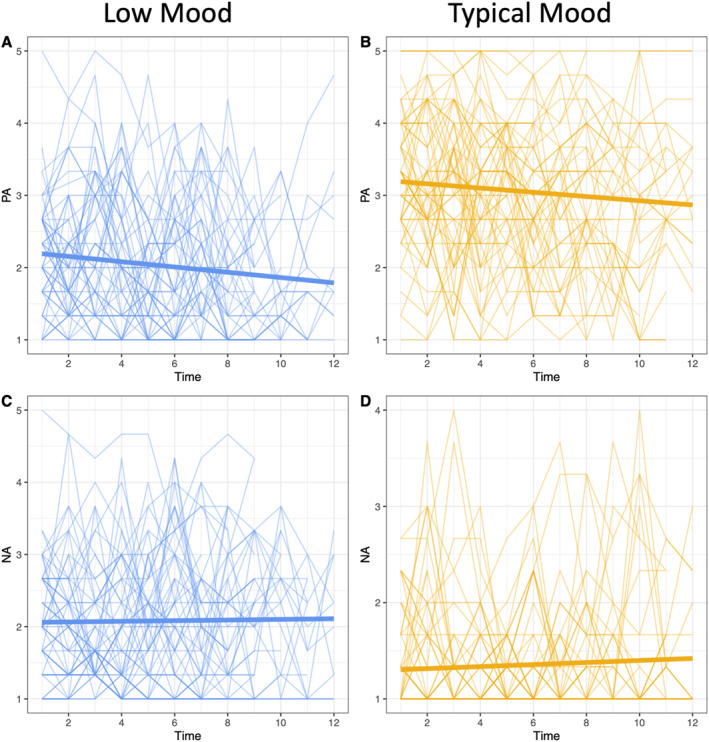
Positive affect (PA) (Top Row) and negative affect (NA) (Bottom Row) scores over time for the Low Mood (Left Column) and Typical Mood (TM) (Right Column) participants. Thicker lines represent group means.

Figure 1 plots PA and NA levels over time for both groups of adolescents. Mean PA was significantly higher in the TM group (mean = 3.03; SD = 0.81) relative to the LM participants (mean = 2.03; SD = 0.59) (*t*(113.6) = 7.6, *p* < .001; Hedges's *g* = 1.40). Mean NA was significantly lower in the TM group (mean = 1.34; SD = 0.39) relative to the LM adolescents (mean = 2.07; SD = 0.69) (*t*(113.6) = −7.2, *p* < .001; Hedges's *g* = 1.31). In addition to comparing mean levels of NA and PA between groups, the collection of longitudinal affect data allows us to disaggregate between‐person from within‐person variance in NA and PA among these adolescents. Intraclass correlation coefficients (ICCs) were 0.58 and 0.57 for NA and PA, respectively, indicating that approximately 57%–58% of the variance in affect was attributable to between‐individual differences and the remainder (42%–43%) was due to variability over time *within* individuals. The mean within‐person correlation between PA and NA was *r* = −0.26 (SD = 0.43), indicating a modest inverse association. PA and NA exhibited a relatively stronger inverse association at the between‐person level (*r* = −0.41) (i.e., those adolescents with relatively high mean PA tend to have relatively lower mean NA).

### Group differences in mind‐wandering

The frequency of mind‐wandering was higher among LM adolescents (55.6%) relative to TM participants (37.6%). This contrast was statistically significant based on the results of the Bayesian logistic regression: the posterior mean of the log odds ratio of mind‐wandering between TM and LM was −0.84 and its posterior 95% credible interval (−1.22, −0.43) did not include zero. For the valence of thoughts during mind‐wandering, LM adolescents were more likely to have unpleasant thoughts (36.8%), relative to pleasant (28.6%) or neutral (34.6%) thoughts. In contrast, TM participants were more likely to mind‐wander to pleasant thoughts (43.9%), relative to unpleasant (20.8%) or neutral (35.3%) thoughts. The results from the Bayesian model confirmed that there was a significant between‐group difference in the odds of unpleasant thoughts during episodes of mind‐wandering (log‐OR for TM versus LM = −0.89, 95% credible interval = [−1.55, −0.23]). See Supporting Information S1 for additional details.

There were no significant between‐group differences in the self‐referential or temporal quality of thoughts during mind‐wandering episodes (see Supporting Information S1). Both groups spent more time mind‐wandering about self‐referential thoughts (LM = 44.9%; TM = 42.2%) relative to thoughts about others (LM = 17.4%; TM = 20.0%) or something else (LM = 37.7%; TM = 37.8%). In addition, both groups spent more time having future‐oriented thoughts (LM = 57.4%; TM = 68.1%) relative to thought about the past (LM = 15.4%; TM = 12.0%) or neither (LM = 27.2%; TM = 19.8%).

### Relation between thought characteristics and affect

**FIGURE 2:**
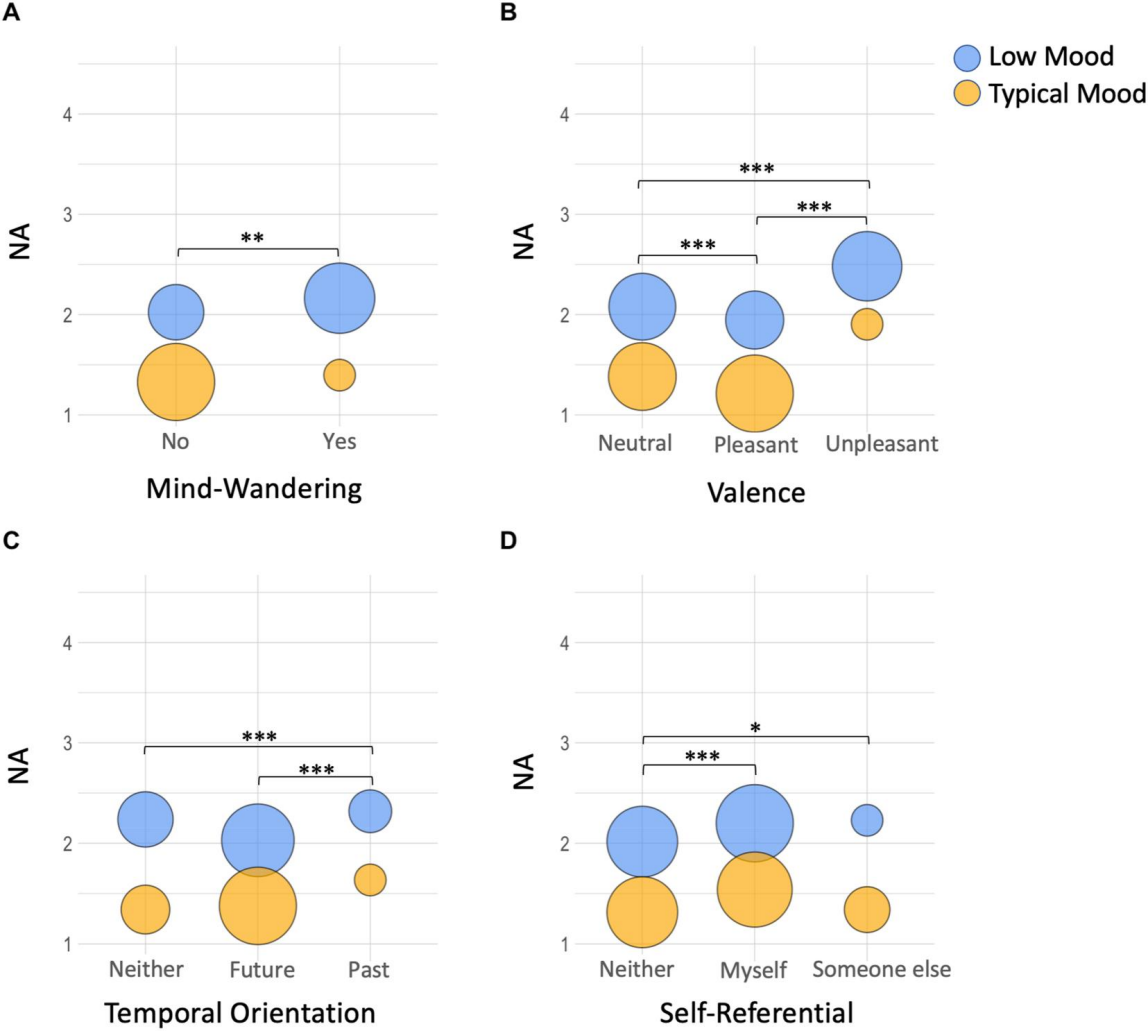
Mean negative affect (NA) for Low Mood (LM) and Typical Mood (TM) groups as a function of mind‐wandering, as well as the valence, temporal orientation, and self‐referential quality of thoughts. Bubble area is proportional to the frequency of ecological momentary assessment (EMA) samples for that group. For example, for the top left panel, the LM group were more likely to report mind‐wandering at EMA timepoints relative to the TM group (55.6% vs. 37.6%, respectively). The bubble sizes are proportional to these percentages. *p < .05; **p < .01; ***p < .001

**FIGURE 3:**
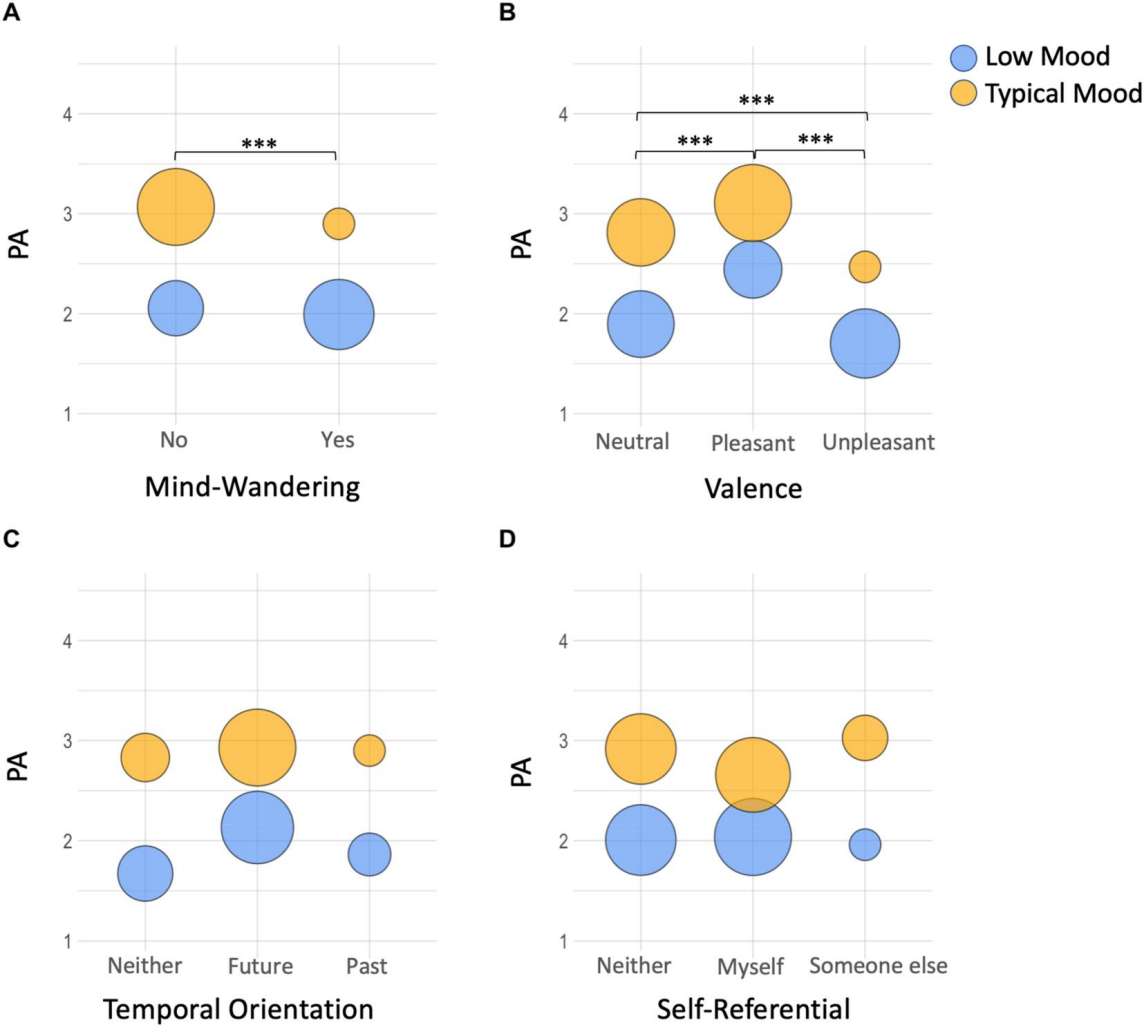
Mean negative affect (NA) for Low Mood (LM) and Typical Mood (TM) groups as a function of mind‐wandering, as well as the valence, temporal orientation, and self‐referential quality of thoughts. Bubble area is proportional to the frequency of ecological momentary assessment samples for that group. *p < .05; **p < .01; ***p < .001

The relationship between each thought characteristic and NA and PA is displayed in Figures 2 and 3, respectively. In univariate analyses (i.e., only including mind‐wandering as a predictor, and no other thought characteristics), mind‐wandering was associated with higher concurrent NA (*b* = 0.11; *t*(962.2) = 2.80, *p* = .005) and lower PA (*b* = −0.20; *t*(962.1) = −3.93, *p* < .001). In addition, lagged analyses revealed that mind‐wandering predicted higher NA (*b* = 0.13; *t*(494.2) = 2.11, *p* = .036) and lower PA (*b* = −0.16; *t*(496.8) = −2.29, *p* = .028) at the next EMA timepoint, but not vice versa (i.e., PA and NA did not predict mind‐wandering at the next timepoint; *p*s > .28). Group (LM vs. TM) did not moderate any of the above concurrent or lagged associations between mind‐wandering and NA or PA (all *p*s > .10), which supports the pooling of these two groups into one analytic sample rather than running separate analyses for each group.

Next, we tested a multivariable model simultaneously including each thought characteristic as predictor variables, which revealed that negatively‐valenced, self‐referential, and past‐oriented thoughts were each associated with higher NA (see Table 2, left panel), even when controlling for plausible confounds (i.e., engagement in a relatively unpleasant activity, being alone vs. with someone, depressive [CESD] symptom severity). As described above, dividing the relevant parameter estimates for each significant predictor by the mean within‐person SD of NA (0.45) provides an estimate of the relative ES of that variable. Unpleasant thoughts were associated with NA values 0.48 higher than pleasant thoughts (ES = 0.48/0.45 = 1.07), a large ES. The effects of self‐referential (ES = 0.24) and past‐oriented thoughts (ES = 0.36) were small to medium in ES terms.

**TABLE 2:** Relation between spontaneous thought characteristics and negative/positive affect

	Negative affect (NA)			Positive affect (PA)		
Predictors	Estimates	95% CI	*p*	Estimates	95% CI	*p*
(Intercept)	1.47	1.27 to 1.67	<.001	2.18	1.94 to 2.42	<.001
Time	0.01	−0.00 to 0.02	.294	−0.03	−0.04 to −0.01	<.001
CESD depression	0.02	0.02 to 0.03	<.001	−0.02	−0.03 to −0.02	<.001
Activity enjoyment	−0.11	−0.14 to 0.08	<.001	0.25	0.21 to 0.29	<.001
With someone (*Yes*)	−0.01	−0.09 to 0.07	.779	0.23	0.13 to 0.32	<.001
Mind‐wandering (*Yes*)	−0.04	−0.11 to 0.04	.383	−0.16	−0.26 to −0.07	.001
Valence (*Pleasant*)	−0.14	−0.22 to −0.06	.001	0.31	0.22 to 0.41	<.001
Valence (*Unpleasant*)	0.34	0.24 to 0.43	<.001	−0.23	−0.34 to −0.12	<.001
Temporal (*Future*)	0.06	−0.03 to 0.14	.174	0.09	−0.00 to 0.19	.062
Temporal (*Past*)	0.16	0.04 to 0.28	.008	0.16	0.03 to 0.30	.021
Self‐referential (*Myself*)	0.11	0.03 to 0.19	.008	0.05	−0.05 to 0.14	.350
Self‐referential (*Someone else*)	0.07	−0.02 to 0.17	.133	0.04	−0.07 to 0.15	.486
Marginal *R* ^2^/Conditional *R* ^2^	0.374/0.653			0.431/0.689		

*Note*: For categorical predictors, parameter estimates are provided for each level of a given predictor relative to the reference level (Mind‐Wandering = No; Valence = Neutral; Temporal = Neither; Self‐Referential = Neither). For example, Temporal (*Past*) has a parameter estimate of 0.16 for the NA model which indicates that thoughts about the past are, on average, associated with NA levels 0.16 points higher than thoughts about neither the past nor future (adjusting for covariates). Marginal *R* ^2^ considers the variance associated with fixed effects, whereas the conditional *R* ^2^ takes both the fixed and random effects into account.

In contrast, task‐related (i.e., *not* mind‐wandering) and positively‐valenced thoughts, as well as past‐oriented thoughts were linked to higher PA (Table 2, right panel). The mean within‐person SD of PA was 0.63, and thus effect sizes were as follows for task‐related thoughts (ES = 0.25), pleasant versus unpleasant thoughts (ES = 0.86), and thoughts about the past (ES = 0.25). There was also a non‐significant trend (*p* = .06) such that future‐oriented thoughts were associated with higher PA (ES = 0.14).

Finally, identical multivariable models were run with lagged affect scores as the dependent variable. No significant relationships emerged in these models (*p*s > .14). See Supporting Information S1 for additional analyses and figures.

## DISCUSSION

Mind‐wandering was common in our adolescent sample, in particular for the LM group (56% of EMA timepoints relative to 38% for TM participants) and was associated with both higher NA and lower PA. This difference may reflect the fact that the LM adolescents (who had elevated depressive symptoms) were more likely to be engaged in negative, ruminative thought. Indeed, as reported above, the LM group were most often mind‐wandering to unpleasant thought content. In addition, the higher rate of mind‐wandering in this group may also be due to broader depression‐related attentional control impairments (Vilgis et al., 2015; Webb, Israel et al., 2021).

Notably, the relationship between mind‐wandering and higher NA was no longer significant when including relevant thought dimensions in our model. Specifically, in the latter model, negatively‐valenced, past‐oriented and self‐focused thoughts were each independently associated with higher concurrent NA, while adjusting for several plausible confounds. Importantly, these effects emerged from a multivariable model within which each thought characteristic was simultaneously included. In other words, both past‐oriented and self‐focused thoughts were significantly associated with elevated NA even when accounting for whether these thoughts were negatively‐valenced or not. Although speculative, it may be that even when adolescents' thoughts shift toward past events that are neutral or pleasant, they may be engaging in subtle comparisons which serve to highlight what is lacking in their lives right now relative to back then, resulting in increased NA. Overall, past‐oriented thoughts were relatively infrequent (LM = 15.4%; TM = 12.0%), but self‐focused thoughts were very common, present at over 40% of EMA timepoints (LM = 44.9%; TM = 42.2%). Prior studies have found past‐oriented thoughts to be associated with worse mood in adult samples (Ruby et al., 2013; Smallwood & O'Connor, 2011). However, we are not aware of any prior studies in adults or youth showing that self‐focused thoughts are associated with worse mood independent of the valence of those thoughts. Given that adolescence is a critical period for self‐concept formation, it may be that self‐focused thoughts often breed judgments or comparisons (e.g., between self and other peers, or current self and ideal/future self) that increase NA in the moment. In addition, although the clinical psychological literature has focused on the effects of *negative* self‐referential thinking (e.g., rumination) on mood, contemplative wisdom traditions—most notably Buddhism—have emphasized the much broader role of self‐referential thought in contributing to unhappiness (Goldstein, 2016; Hadash et al., 2016). Indeed, there is emerging evidence that changes in self‐referential thinking may be one key mechanism through which mindfulness training exerts its beneficial effects on mood and internalizing symptoms (Lin et al., 2018), and that mindfulness training—with its emphasis on cultivating nonjudgmental present‐moment awareness—is most beneficial for those adolescents with a greater tendency towards maladaptive self‐referential thinking patterns (Webb et al., 2022; Webb, Swords et al., 2021).

In contrast to the NA findings, mind‐wandering remained significantly associated with lower PA, even after adjusting for other thought characteristics and plausible confounding variables. In other words, thinking about something other than what one is currently doing is still significantly associated with lower PA even after controlling for the effect of thought valence, temporal orientation, and self‐referential quality. It may be that focusing attention on the task at hand is more likely to promote positive affective states (e.g., stimulate interest in the current activity or promote savoring and experiential pleasure) than to reduce negative emotional states (Blanke et al., 2018). Being fully attentive to what one is currently doing may promote interest and pleasure in an activity, but may also inhibit task‐unrelated thoughts that interfere with PA. Alternatively, low levels of PA (e.g., boredom) may promote greater mind‐wandering.

The association between past‐oriented thoughts and greater PA was unexpected and seemingly contradicts the abovementioned finding that thoughts about the past were associated with higher NA. However, PA and NA are only modestly inversely correlated within individuals (*r* = −0.26 in the present study). In other words, elevations in NA do not necessarily correspond with decreases in PA, and both elevated NA and PA can co‐occur simultaneously. For example, an adolescent could be thinking about a past event that simultaneously increases anxiety (i.e., higher NA), as well as interest or excitement (i.e., higher PA). It should also be noted that in univariate analyses (i.e., not including covariates; see Figure 3) thoughts about the past were not significantly related to PA. There was also a non‐significant trend of future‐oriented thoughts, which were very common (present at well over half of EMA timepoints; LM = 57.4%; TM = 68.1%), being associated with higher PA. With regards to covariates, it also worth noting that being with someone (relative to being alone) at the time of the EMA survey was significantly associated with higher PA but not lower NA (see Table 2).

The concurrent associations between spontaneous thought characteristics and worse affect could be due to the former causing the latter and/or vice versa (or be due to a third variable confound not included in our models). Lagged analyses were conducted to test the directionality of these associations. In a multivariable model (i.e., simultaneously including all thought characteristics and covariates) no lagged associations were significant (lagged associations were detected in a univariate model with mind‐wandering predicting higher NA and lower PA at the next EMA timepoint, but NA/PA were not associated with future mind‐wandering). There are a few EMA studies—primarily conducted in adult samples—that have tested lagged associations between mind‐wandering and affect (or related emotional well‐being constructs). Some of these studies provide support for mind‐wandering predicting future mood states (e.g., Killingsworth & Gilbert, 2010; Ruby et al., 2013; Welz et al., 2018), whereas others have found support for the reverse association, or a bidirectional relation (e.g., Webb, Israel et al., 2021). It is important to note that, in the present study, the average length of time between EMA surveys was approximately 3 h. It may be that a denser EMA sampling strategy, with more frequent surveys per day, is required to capture the short‐term impact of thought characteristics on affect and vice versa. In addition, future studies may benefit from a longer EMA data collection period (e.g., 30 days). However, when designing such a study, researchers must be very mindful to not overburden adolescent participants, which could negatively impact survey compliance, or lead to stereotyped or random responding to questions.

As an alternative to the limitations of observational EMA studies, experimental inductions can also be used to test the directionality of the relationship between different spontaneous thought characteristics and affect in adolescents. For example, studies have found that negative mood inductions increase mind‐wandering (Marcusson‐Clavertz et al., 2020; Seibert & Ellis, 1991; Smallwood et al., 2009), with some limited evidence that inducing mind‐wandering may increase negative moods (Gibb et al., 2022). Of relevance, there is a substantial body of research showing that experimentally inducing negatively‐valenced categories of mind‐wandering focused on the past (i.e., rumination inductions; e.g., Donaldson & Lam, 2004; Lavender & Watkins, 2004; Nolen‐Hoeksema & Morrow, 1993) or future (i.e., worry inductions; e.g., Frala et al., 2014; Llera & Newman, 2010) cause worse mood. Future studies could consider experimentally inducing specific facets of mind‐wandering (e.g., self‐referential thoughts vs. mind‐wandering unrelated to the self) and assess impacts on PA versus NA. In summary, the relationship between mind‐wandering and affect is likely bidirectional, moderated by content (e.g., valence of thoughts), and time‐limited (i.e., the effect of spontaneous thought on affect, and vice versa, may dissipate more quickly than can be captured by the EMA sampling frequencies used in most studies).

The present study was part of a larger project focused on recruiting adolescents with elevated anhedonia (LM group) and a control group (TM group). The inclusion/exclusion criteria were such that the LM group had elevated anhedonia and overall depressive symptoms, and a number of LM participants had MDD and/or anxiety disorders, as these were not exclusionary. In contrast, the TM group were free of any psychiatric diagnoses and had low levels of depressive symptoms and anhedonia. Although these inclusion/criteria ultimately provided meaningful variance in our predictor and outcome variables (e.g., see Figure 2), the inclusion of two relatively bimodal groups may have increased the chances of identifying group differences. Future studies could examine the extent to which the present findings are replicated using alternative inclusion/exclusion criteria.

This study had several limitations. First, other characteristics of spontaneous thought, which were not assessed, may influence affect, such as meta‐awareness of mind‐wandering states or whether mind‐wandering is intentional or unintentional (Seli et al., 2016, 2019; Vannucci et al., 2020). Relatedly, given evidence that sleep disturbances increase during adolescence (Donskoy & Loghmanee, 2018; Lemola et al., 2015; Wheaton, 2018) and predict greater mind‐wandering (Marcusson‐Clavertz et al., 2020), research is needed to investigate the dynamic interplay between sleep, mind‐wandering and affective disturbance in teens. In addition, a denser EMA sampling strategy, with more frequent surveys per day, may have revealed significant lagged relations between thought characteristics and affect. Finally, the sample consisted of predominantly non‐Hispanic White adolescents, limiting generalizability. These limitations notwithstanding, the present findings contribute to a currently limited but growing body of literature on the relationship between spontaneous thought characteristics and affect in adolescents. If replicated, these findings could inform more nuanced assessments of and targeted interventions for specific dimensions of mind‐wandering which contribute to heightened NA versus blunted PA in teens.

## AUTHOR CONTRIBUTIONS

**Christian A. Webb**: Conceptualization; Data curation; Formal analysis; Funding acquisition; Investigation; Methodology; Project administration; Supervision; Visualization; Writing – original draft; Writing – review & editing. **Anna O. Tierney**: Data curation; Formal analysis; Project administration; Writing – original draft; Writing – review & editing. **Hallie A. Brown**: Data curation; Investigation; Methodology; Writing – original draft; Writing – review & editing. **Erika E. Forbes**: Conceptualization; Funding acquisition; Supervision; Writing – review & editing. **Diego A. Pizzagalli**: Funding acquisition; Investigation; Methodology; Supervision; Writing – review & editing. **Boyu Ren**: Formal analysis; Visualization; Writing – original draft; Writing – review & editing.

## CONFLICT OF INTEREST

Christian A. Webb serves on the JCPP *Advances* Editorial Advisory Board. Over the past 3 years, Dr. Pizzagalli has received consulting fees from Albright Stonebridge Group, BlackThorn Therapeutics, Boehringer Ingelheim, Compass Pathway, Concert Pharmaceuticals, Engrail Therapeutics, Neuroscience Software, Neurocrine Biosciences, Otsuka Pharmaceuticals, and Takeda Pharmaceuticals, as well as one honorarium from the Psychonomic Society (for editorial work) and Alkermes. In addition, he has received stock options from BlackThorn Therapeutics and Compass Pathways, as well as research support from National Institute of Mental Health, Dana Foundation, Brain and Behavior Research Foundation, and Millennium Pharmaceuticals. The remaining authors have declared that they have no competing or potential conflicts of interest.

## ETHICAL CONSIDERATIONS

The study was approved by the Mass General Brigham IRB. Written informed consent was required from participating parents and children provided assent (18 year olds provided consent).

## Supporting information

Supporting Information S1

## Data Availability

The data that support the findings of this study are available from the corresponding author upon reasonable request.
