# Supplementary material for: Spontaneous thought characteristics are differentially related to heightened negative affect versus blunted positive affect in adolescents: An experience sampling study
Source: JCPP Adv. 2022 Nov 22;2(4):e12110. doi: 10.1002/jcv2.12110 (PMC9937432; doi:10.1002/jcv2.12110)

**Supporting Information**

**Spontaneous Thought Characteristics are Differentially Related to Heightened Negative Affect vs. Blunted Positive Affect in Adolescents: An Experience Sampling Study**

**Supplemental Methods**

**Participants**

Depressive (CES-D) symptom scores ranged from none to severe (0 – 51), and were significantly higher in LM adolescents (mean = 33.4; SD = 9.9) relative to the TM group (mean = 5.8; SD = 5.03)(*t*(84.85, Satterthwaite for unequal variances) = 18.98, *p* < .001; Hedges’s *g* = 3.50). Similarly, anhedonia (SHAPS) scores ranged from 14 – 48, and were significantly higher in LM participants (mean = 34.43; SD = 5.8) relative to the TM group (mean = 18.53; SD = 4.51)(*t*(107.7, Satterthwaite for unequal variances) = 16.53, *p* < .001; Hedges’s *g* = 3.05.

**Self-Report Measures**

**Measures**

**The Snaith-Hamilton Pleasure Scale (SHAPS).** The SHAPS is a 14-item scale designed to measure anhedonia, with higher scores indicating greater anhedonic severity (Snaith et al., 1995). It includes items such as “I would enjoy being with my close family and friends,” and “I would feel pleasure when I receive praise from other people.” Participants indicated the degree to which they agreed with each statement on a 4-point Likert scale, with 1 corresponding to “strongly disagree,” and 4 corresponding to “strongly agree.” The SHAPS total score (dimensional scoring; range 14-56) demonstrated excellent internal consistency in this sample (α = 0.95).

**Center for Epidemiological Studies Depression Scale (CES-D).** The CES-D is a 20-item scale designed to measure symptoms of depression, with higher scores indicating greater depressive symptom severity (Radloff, 1977). It includes items such as “I was bothered by things that usually don’t bother me,” and “I talked less than usual.” Participants indicated the extent to which they experienced different depressive symptoms in the past week on a 4-point Likert scale, with 1 corresponding to “rarely or none of the time (less than 1 day)” and 4 corresponding to “most or all of the time (5 - 7 days).” The CES-D total score demonstrated excellent internal consistency in this sample (α = 0.95).

**Ecological Momentary Assessment (EMA) Split-Half Reliability**

Split-half reliability for the positive affect (PA) and negative affect (NA) EMA measures was computed by randomly splitting the dataset into two halves (by participant ID), computing participant PA and NA means separately for each half of the data and correlating these mean scores.

**Data Analytic Plan**

To test group differences in categorical outcomes (i.e., mind-wandering [yes vs. no], valence [pleasant, unpleasant, vs. neutral thoughts], temporal orientation [thoughts about the past, future vs. present/neither], and self-referential quality [thoughts about the self, others, vs. neither]) we used a Bayesian hierarchical multinomial logistic model implemented in the R (Vers. 4.1.0) package *brms* (Bürkner, 2017)*.* This model can accommodate categorical outcomes with at least two levels. Each outcome was analyzed separately, and group label (LM vs. TM) was the only predictor in the model. We specified random intercepts specific to each subject and a fixed effect of group. Details on the Bayesian prior specification can be found in Bürkner (2017). The posterior distributions of the regression coefficients were approximated using Markov Chain Monte Carlo (MCMC) algorithms in Stan (Carpenter et al., 2017). One MCMC chain with 5,000 iterations was generated and only the last 2,500 iterations were saved for inference.

**Supplemental Results**

**Group Differences in Mind-Wandering**

Within the LM group, there was no significant difference between the probabilities of pleasant/unpleasant thoughts vs. neutral thoughts: posterior mean of log-odds for pleasant vs. neutral = -0.16 [95% credible interval = (-0.59,0.25)]; posterior mean of log-odds for unpleasant vs. neutral = 0.17 [95% credible interval = (-0.25,0.57)]. In contrast, within the TM group, the probability of unpleasant thoughts was significantly lower than neutral thoughts [posterior mean = -0.89+0.17=-0.72, 95% credible interval = (-1.30,-0.21)], whereas the probability of pleasant thought was not significantly different from neutral thoughts [posterior mean = 0.56-0.16=0.4, 95% credible interval = (-0.02,0.80)].

There were no significant between-group differences in the self-referential (log-OR for TM vs. LM for thoughts about the self [posterior mean = -0.21 [95% credible interval = (-0.93,0.49)] or others [posterior mean = 0.23 [95% credible interval = (-0.38,0.82)]) or temporal quality (log-OR for TM vs. LM for thoughts about the past [posterior mean = 0.08 [95% credible interval = (-0.56,0.76)] or future [posterior mean = 0.54 [95% credible interval = (-0.09,1.21)]) of thoughts during mind-wandering episodes.

**Additional Analyses**

Supplemental Table S1 is the same as Table 2 in the main text but adding PA/NA as a covariate.

In response to an anonymous reviewer, we re-reran the multivariable NA model using a log-transform (due to a positive skew for NA) which yielded the same pattern of findings as those reported in Table 2. Specifically, negatively-valenced, past-oriented and self-referential thoughts predicted higher NA (*ps* < .03).

**Reference:**

Bürkner, P.-C. (2017). brms: An R package for Bayesian multilevel models using Stan. *Journal of Statistical Software*, *80*, 1–28.

Carpenter, B., Gelman, A., Hoffman, M. D., Lee, D., Goodrich, B., Betancourt, M., Brubaker, M., Guo, J., Li, P., & Riddell, A. (2017). Stan: A Probabilistic Programming Language. *Journal of Statistical Software*, *76*(1). https://doi.org/10.18637/jss.v076.i01

Radloff, L. S. (1977). The CES-D Scale A Self-Report Depression Scale for Research in the General Population. *Applied Psychological Measurement*, *1*(3), 385–401. https://doi.org/10.1177/014662167700100306

Snaith, R. P., Hamilton, M., Morley, S., Humayan, A., Hargreaves, D., & Trigwell, P. (1995). A scale for the assessment of hedonic tone the Snaith-Hamilton Pleasure Scale. *The British Journal of Psychiatry*, *167*(1), 99–103. https://doi.org/10.1192/bjp.167.1.99

Webb, C. A., Israel, E. S., Belleau, E., Appleman, L., Forbes, E. E., & Pizzagalli, D. A. (2021). Mind-Wandering in Adolescents Predicts Worse Affect and Is Linked to Aberrant Default Mode Network–Salience Network Connectivity. *Journal of the American Academy of Child & Adolescent Psychiatry*, *60*(3), 377–387. https://doi.org/10.1016/j.jaac.2020.03.010

***Table S1.*** Relation between Spontaneous Thought Characteristics and Negative/Positive Affect

|  | **Negative Affect (NA)** | | | **Positive Affect (PA)** | | |
| --- | --- | --- | --- | --- | --- | --- |
| *Predictors* | *Estimates* | *95% CI* | *p* | *Estimates* | *95% CI* | *p* |
| (Intercept) | 1.69 | 1.46 – 1.92 | **<0.001** | 2.40 | 2.14 – 2.66 | **<0.001** |
| Time | 0.00 | -0.01 – 0.01 | 0.601 | -0.03 | -0.04 – -0.01 | **<0.001** |
| PA/NA | -0.10 | -0.15 – -0.05 | **<0.001** | -0.15 | -0.22 – -0.07 | **<0.001** |
| CESD Depression | 0.02 | 0.01 – 0.03 | **<0.001** | -0.02 | -0.03 – -0.01 | **<0.001** |
| Activity Enjoyment | -0.08 | -0.12 – -0.05 | **<0.001** | 0.23 | 0.19 – 0.27 | **<0.001** |
| With Someone *[Yes*] | 0.01 | -0.07 – 0.09 | 0.735 | 0.22 | 0.13 – 0.32 | **<0.001** |
| Mind-Wandering [*Yes*] | -0.05 | -0.13 – 0.03 | 0.213 | -0.16 | -0.25 – -0.07 | **0.001** |
| Valence [*Pleasant*] | -0.11 | -0.19 – -0.03 | **0.008** | 0.29 | 0.20 – 0.39 | **<0.001** |
| Valence [*Unpleasant*] | 0.31 | 0.22 – 0.40 | **<0.001** | -0.19 | -0.30 – -0.08 | **0.001** |
| Temporal [*Future*] | 0.07 | -0.01 – 0.15 | 0.103 | 0.09 | -0.00 – 0.19 | 0.056 |
| Temporal [*Past*] | 0.18 | 0.06 – 0.29 | **0.003** | 0.19 | 0.05 – 0.33 | **0.007** |
| Self-Referential [*Myself*] | 0.11 | 0.03 – 0.19 | **0.006** | 0.06 | -0.04 – 0.16 | 0.221 |
| Self-Referential [*Someone else*] | 0.08 | -0.02 – 0.17 | 0.112 | 0.05 | -0.06 – 0.16 | 0.373 |
| Marginal R^2^ / Conditional R^2^ | 0.383 / 0.668 | | | 0.431 / 0.699 | | |

*Note:* For the predictor variable PA/NA, PA is the predictor for the model in which NA is the outcome, and vice versa. For categorical predictors, parameter estimates are provided for each level of a given predictor relative to the reference level (Mind-Wandering = No; Valence = Neutral; Temporal = Neither; Self-Referential = Neither). For example, Temporal [*Past*] has a parameter estimate of 0.16 for the NA model which indicates that thoughts about the past are, on average, associated with NA levels 0.16 points higher than thoughts about neither the past nor future (adjusting for covariates). Marginal R^2^ considers the variance associated with fixed effects, whereas the conditional R^2^ takes both the fixed and random effects into account.

**Figure Captions**

***Supplemental Figure 1.*** Half violin plots (with jittered datapoints) displaying negative affect (NA) scores as a function of mind-wandering, as well as the valence, temporal orientation, and self-referential quality of thoughts. *p < .05; **p < .01; ***p < .001.

***Supplemental Figure 2.*** Half violin plots (with jittered datapoints) displaying negative affect (PA) scores as a function of mind-wandering, as well as the valence, temporal orientation, and self-referential quality of thoughts.*p < .05; **p < .01; ***p < .001.


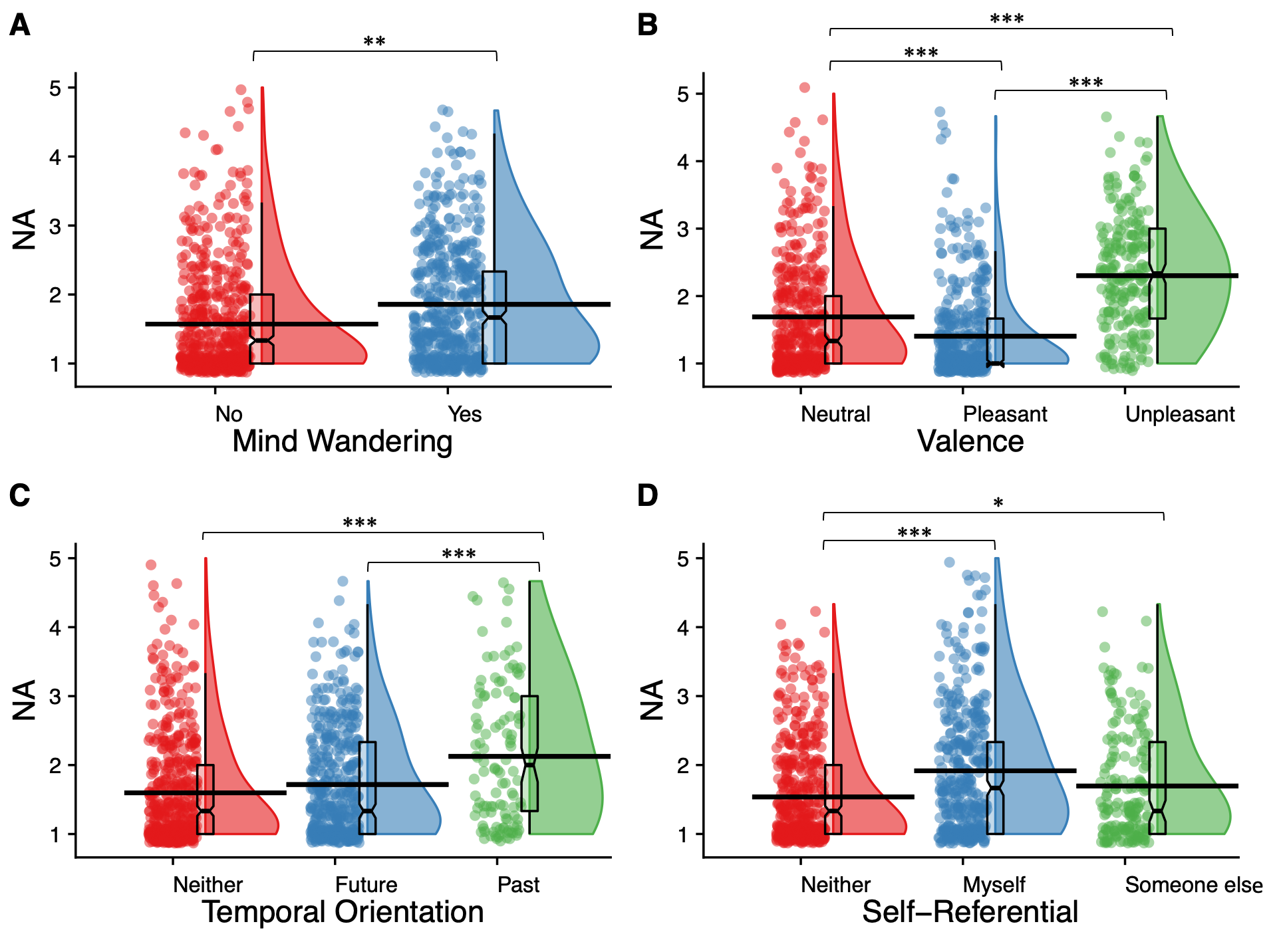


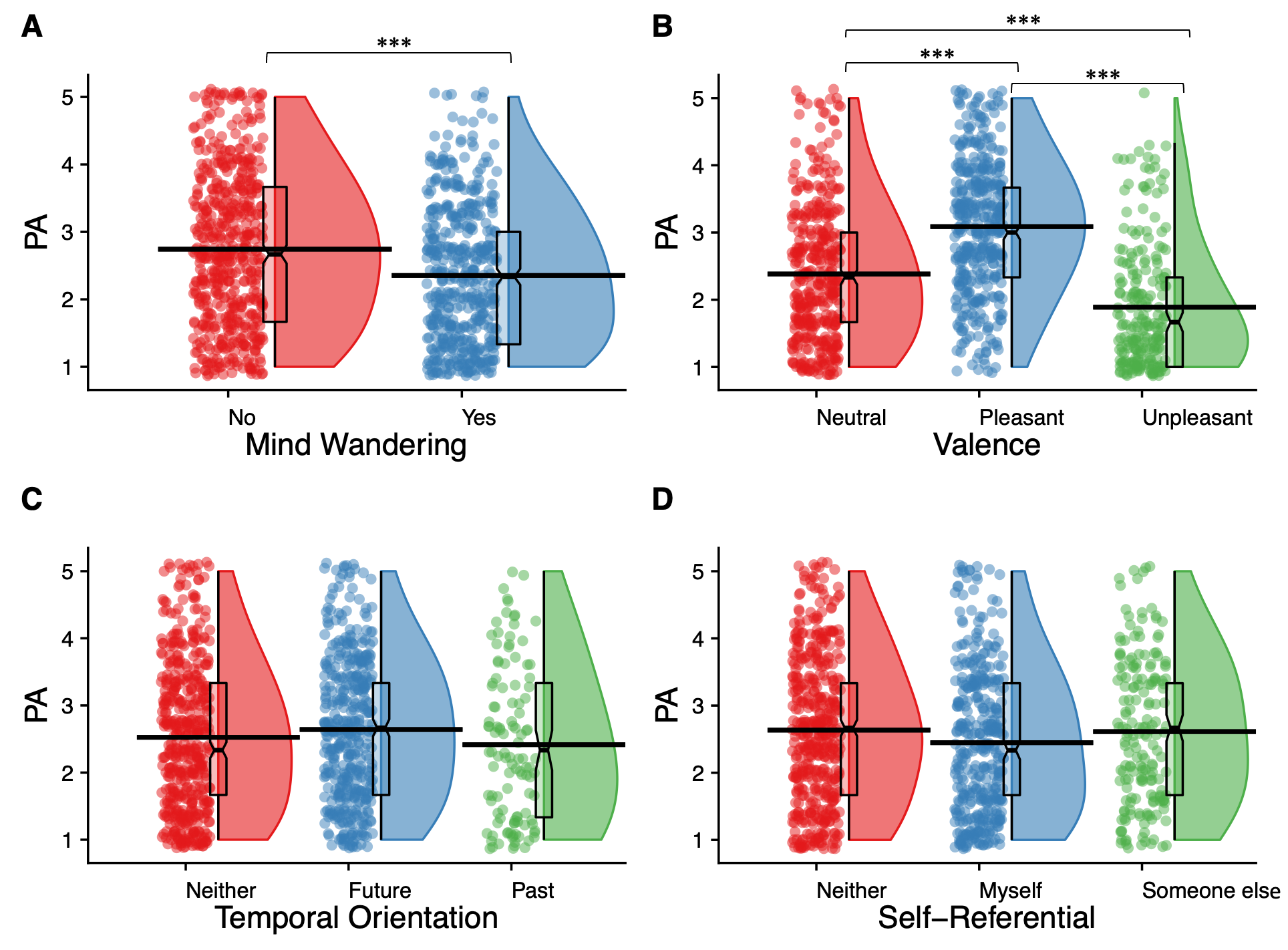

Supplement: Supplementary file 1 — Supporting Information S1 [file JCV2-2-e12110-s001.docx]
